# Criminal Victimisation in People with Severe Mental Illness: A Multi-Site Prevalence and Incidence Survey in the Netherlands

**DOI:** 10.1371/journal.pone.0091029

**Published:** 2014-03-07

**Authors:** Astrid M. Kamperman, Jens Henrichs, Stefan Bogaerts, Emmanuel M. E. H. Lesaffre, André I. Wierdsma, Razia R. R. Ghauharali, Wilma Swildens, Yolanda Nijssen, Mark van der Gaag, Jan R. Theunissen, Philippe A. Delespaul, Jaap van Weeghel, Jooske T. van Busschbach, Hans Kroon, Linda A. Teplin, Dike van de Mheen, Cornelis L. Mulder

**Affiliations:** 1 Epidemiological and Social Psychiatric Research Institute, Department of Psychiatry, Erasmus Medical Centre, Rotterdam, the Netherlands; 2 Department of Developmental Psychology, University of Tilburg, Tilburg, the Netherlands; 3 FPC Kijvelanden, Karid, Roterdam, the Netherlands; 4 Department of Biostatistics, Erasmus Medical Centre, Rotterdam, the Netherlands; 5 L-Biostat, Catholic University Louvain, Louvain, Belgium; 6 Dutch Crime and Victim Survey Desk, The Hague, the Netherlands; 7 Altrecht Mental Health Care, Utrecht, the Netherlands; 8 Dijk en Duin Mental Health Centre, Parnassia Group, Castricum, the Netherlands; 9 Parnassia Psychiatric Institute, The Hague, the Netherlands; 10 VU University and EMGO Institute for Health and Care Research, Amsterdam, the Netherlands; 11 GGZinGeest Mental Health Care, Amsterdam, the Netherlands; 12 Department of Psychiatry, VU Medical Center, Amsterdam, the Netherlands; 13 Department of Psychiatry and Psychology, School of Mental Health and Neuroscience, Maastricht University Medical Centre, Maastricht, the Netherlands; 14 Mondriaan Mental Health Care, Heerlen, the Netherlands; 15 Department Tranzo, Tilburg School of Social and Behavioral Sciences, University of Tilburg, Tilburg, the Netherlands; 16 Department of Psychiatry, University Medical Center Groningen, University of Groningen, Groningen, the Netherlands; 17 Department of Movement and Education, Windesheim University of Applied Sciences, Zwolle, the Netherlands; 18 Trimbos Institute, Utrecht, the Netherlands; 19 Department of Psychiatry and Behavioral Sciences, Feinberg School of Medicine, Northwestern University, Chicago, Illinois, United States of America; 20 IVO Addiction Research Institute, Rotterdam, the Netherlands; 21 Erasmus Medical Centre, Rotterdam, the Netherlands; 22 Department of Health Promotion, Maastricht University, Maastricht, the Netherlands; 23 BavoEuropoort, Rotterdam, the Netherlands; Chiba University Center for Forensic Mental Health, Japan

## Abstract

**Background:**

Although crime victimisation is as prevalent in psychiatric patients as crime perpetration (and possibly more so), few European figures for it are available. We therefore assessed its one-year prevalence and incident rates in Dutch severely mentally ill outpatients, and compared the results with victimisation rates in the general population.

**Method:**

This multisite epidemiological survey included a random sample of 956 adult severely mentally ill outpatients. Data on victimisation were obtained using the victimisation scale of the Dutch Crime and Victimisation Survey, which assesses crime victimisation over the preceding 12 months. Comparison data were derived from the nationwide survey on safety and victimisation in the Netherlands. Prevalence and incident rates were weighted for sex, age, ethnicity and socioeconomic status, and compared with a general population sample matched by region (N = 38,227).

**Results:**

In the past year, almost half of the severely mentally ill outpatients (47%) had been victim of a crime. After control for demographic differences, prevalence rates of overall and specific victimisation measures were significantly higher in severely mentally ill outpatients than in the general population. The relative rates were especially high for personal crimes such as violent threats (RR = 2.12, 95% CI: 1.72–2.61), physical assaults (RR = 4.85, 95% CI: 3.69–6.39) and sexual harassment and assaults (RR = 3.94, 95% CI: 3.05–5.09). In concordance, severely mentally ill outpatients reported almost 14 times more personal crime incidents than persons from the general population (IRR = 13.68, 95% CI: 12.85–14.56).

**Conclusion:**

Crime victimisation is a serious problem in Dutch severely mentally ill outpatients. Mental-healthcare institutions and clinicians should become aware of their patients’ victimisation risk, and should implement structural measures to detect and prevent (re-)victimisation.

## Introduction

Most earlier psychiatric studies addressing crime and violence focused on patients with severe mental illness (SMI) as perpetrators [1], [2]. Patients with SMI are often perceived as dangerous and unpredictable and more prone to perpetrating violence than those in the general population [3]–[7].

Few studies have examined the risk of crime victimisation (i.e. various types of property and personal crime victimisation) among people with SMI in outpatient care [1]. A randomised survey in Chicago among 936 adult SMI outpatients showed that even after correction for demographic difference, the prevalence of personal crime victimisation in these individuals was 11 times higher than in the general population [8]. A literature review also showed that crime victimisation rates among US psychiatric outpatients were much higher than rates of crime perpetration [1]. A systematic review based on nine studies reported that the prevalence rates of crime victimisation among patients with SMI ranged from 4% to 35% [9]. A recent European study among involuntary admitted patients showed that 28% of a mixed European, and 38% of an English patient group had been victim of physical violence in the year prior to their admission [10].

SMI patients are commonly diagnosed with psychotic, bipolar, or major depressive disorders [11]. Due not only to psychological problems such as impulsiveness, substance abuse, poor reality testing and judgment, but also to impaired social skills, they probably constitute a high-risk group for victimisation. Overall conditions – such as unemployment, poverty, homelessness, and conflicted relationships – can contribute to the risk of victimisation [12]–[21].

In European countries such as the Netherlands, research on crime victimisation among SMI patients is largely absent, although there is no clear reason for this [9]. While deinstitutionalisation has been less drastic in the Netherlands than in the United States [22], most Dutch SMI patients are no longer in the protective care of 24-hour hospital services: about 90% of SMI patients receive outpatient care and/or are living in supported-housing facilities (e.g. halfway houses) in close contact with the community. The extent of homelessness among SMI persons in Holland is smaller than in the United States as a result of the Dutch welfare system [23].

While previous research has often examined the prevalence of crime victimisation [9], very few studies have investigated the number of incidents per 1,000 people in the preceding 12 months. By studying both prevalence and incident rates, one gains better insight into the extent of the victimisation within the population of victims e.g. whether a person was victim of a single or multiple incidents. The current study is the first nationwide multi-site epidemiological study in Europe to establish not only the 12-month prevalence of crime victimisation among SMI outpatients relative to rates in the general population (i.e. the proportion of subjects affected by it), but also its 12-month incident rate (i.e. the number of incidents per 1,000 people over one year).

## Method

### Design

This study was approved by the Medical Ethics Committee at Erasmus Medical Centre, Rotterdam (MEC-2010-232). Written informed consent was obtained from all participants. We did not make use of surrogate consent procedures. Compromised ability to consent, as determined by their primary clinician, was regarded as an exclusion criterion.

This study is embedded in the Victimisation in Psychiatric Patients study, a cross-sectional epidemiological survey of a large random community sample of patients with SMI in the Netherlands. Participants were randomly selected from the caseload of six Mental Healthcare (MHC) institutions in the Netherlands that provide outpatient care to patients suffering from SMI. Located in urban and rural areas of the Netherlands, these institutions provide care to a range of 240 to 2,000 patients (approx. 9,250 patients in total) with chronic (≥2 year duration) psychotic, bipolar or major depressive disorders. Accurate and comprehensive nationwide registration of MHC is lacking, therefore exact figures on the number of SMI patients in the Netherlands are missing. Recent estimations range from 64,000 to 160,000 SMI patients nationwide, of which 56% are in outpatient treatment [24], [25]. In terms of diagnosis, MHC use, and demographic characteristics, the patient populations at these institutions are representative of the chronic psychiatric patient population in the Netherlands [24]. Participants were enrolled in the study between December 2010 and April 2012.

### Participants

Outpatients aged 18 to 65 years at one of the MHC institutions were eligible for the study. For inclusion, they had to have been diagnosed (by the treating psychiatrist using a clinical interview) with a chronic (duration ≥2 year) psychotic, bipolar or major depressive disorder, according to DSM-IV-TR criteria. Outpatients with psycho-organic disorders were excluded, as were those with insufficient command of the Dutch language and those whose psychiatric condition as determined by their primary clinician (severe symptomatology, high levels of aggression or cognitive impairments), prevented them from answering study questions or consenting with the interview.

### Procedures

A random sample of 3,336 potentially eligible outpatients was selected from the patient administration system at each participating institution based on information available in the electronic patient files (EPF). The inclusion and exclusion criteria obtained from the EPF were then checked and crosschecked by each primary MHC clinician, who was responsible for treating the patient in question and for coordinating this treatment. In most cases this was a psychiatric nurse. This procedure resulted in a eligible sample of 2,572 patients.

Eligible patients received an invitation letter explaining the study procedure and confidentiality issues; it also contained a refusal form that could be returned free of charge. Two weeks after dispatch of the letter, patients who had not returned the refusal form were contacted by the interviewers for verbal confirmation of their willingness to participate. A face-to-face interview was scheduled with those who agreed. Data on crime victimisation were collected as part of a structured computer-assisted interview by trained interviewers with a Master’s degree in the social sciences. Next to crime victimisation, this interview consisted of questions on police contact, juridical and personal consequences of the reported victimisation incidents, discrimination, self-stigmatisation, and a range of potential risk factors i.e. violent perpetration, posttraumatic stress symptoms, drug- and alcohol abuse, psychosocial functioning, victimisation in early childhood, problems with regards to aggression-regulation, and sociodemographic characteristics. These data will be presented in future papers. Each respondent received a €20 cash incentive at the end of the interview. On average, the patient interview took 75 minutes (range: 40–160 minutes), and was carried out at the respondent’s discretion in his or her home or MHC institution.

**Figure 1:**
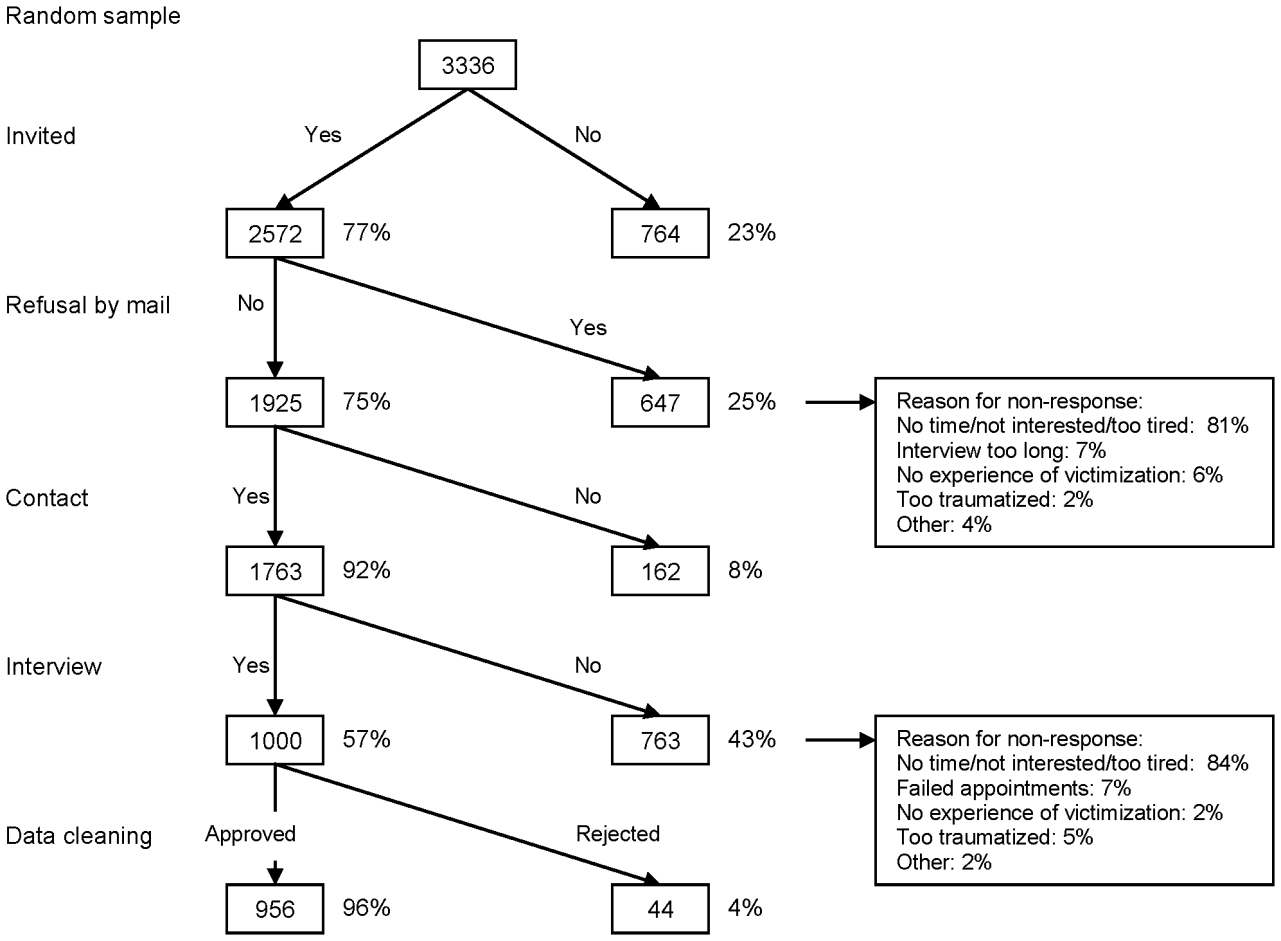
Data-acquisition flowchart.

Invitation letters were sent to 2,572 patients. Twenty-five percent of the invitees (N = 647) had returned a refusal form; no further attempts were made to contact them. The remaining patients were first approached by telephone. If three or more calls were unanswered or a telephone number was incorrect, a reminder letter was sent (9% of cases), the primary clinician was involved (13% of cases), and a final house call was paid (6% of cases). On average, three attempts were made to contact a patient (range 1–11). Despite the attempts to contact them, 8% of the patients could not be reached. Of the remaining patients, 763 (43%) refused to participate. A thousand interviews were conducted. After data cleaning, the interviewer judged 44 interviews (4%) to be unreliable (i.e. to contain severely inconsistent or aberrant answers) due to the respondent’s severe psychiatric symptomatology (i.e. delusions, hallucinations, or cognitive impairments). Since this was an exclusion criterion, the interviews were removed from the sample and were not included in the non-response analyses. In total, we interviewed 37% of all patients invited (956/2,572), and 54% of all those contacted (956/1,763). This resulted in 956 SMI outpatients who were interviewed on crime victimisation. Figure 1 depicts the flow chart of the data acquisition.

Response analyses were performed using demographic and clinical information extracted from the EPF at the MHC institutions (i.e. sex, age, ethnicity, marital status, primary diagnosis, and psychiatric hospital admission or admissions in the past year). The socioeconomic and demographic characteristics of a patient’s neighbourhood (i.e. income levels, population density, and unemployment rate) for 2010 were obtained from Statistics Netherlands, i.e. the national bureau of statistics.

The random sample (N = 3,336) was compared with the eligible and invited patients (N = 2,572); patients contacted (N = 2,366) were compared with the patients who had been unreachable (N = 162); and responders (N = 956) were compared with non-responders (N = 1,572). The analyses showed no significant differences between the random and invited samples. Analyses between contacted and unreachable patients showed that unreachable patients were an average of 1.7 year younger (t(2526) = 2.016; p<0.05), earned 500 Euro less per year (t(2291) = 2.299; p<0.05), and were more likely to have been admitted to a psychiatric hospital or to have admission information missing from their files (χ^2^(2) = 13.379; p<0.05). Responders and non-responders did not differ with regard to any of the characteristics mentioned above.

Finally, we used multivariate logistic regression analyses to identify any overall effect and interaction effects of demographic, clinical, neighbourhood social-economic variables, and participating MHC institution for contact and response rates. With regard to the contact rate, it was more difficult to contact low or lower-income patients at two of the six MHC institutions (χ^2^(34) = 69.837; p<.001). With regard to the response rate, the model did not fit the data, not indicating potential bias in our sample by demographic, clinical, neighbourhood social-economic variables, or participating MHC institution (χ^2^(34) 39.319; p = .244).

### Instruments

To establish the twelve-month prevalence and 12-month incident rates of crime victimisation, we used the crime victimisation scale of the Dutch Crime and Victimisation Survey (in Dutch: *Integrale Veiligheidsmonitor*) (IVM) [26]. This strongly resembles the International Crime Victimization Survey [27], which consists of 14 screening questions on being a victim of one or more of the following: burglary, attempted burglary, bicycle theft, car theft, theft from car, car vandalism, pick-pocketing, robbery, theft (other than previously categorised), vandalism (other than previously categorised), sexual harassment or assault, threats of violence, physical assault, or crime (other than previously categorised). For each incident reported in the preceding 12 months, it assesses detailed information. These detailed data allow the researcher to determine whether the event is a crime, when and where it occurred, who was involved, whether the police was notified, whether there was property loss, and the degree of physical injury. To minimise the effect of telescoping, the respondents are asked to recall incidents over the past five years before they are asked to recall incidents over the past 12 months. There are no traditional reliability and validity scores for the IVM crime-victimisation scale [26].

### Comparison Group

Comparison data were derived from the most recent IVM survey, an annual survey on safety, quality of life, and crime victimisation among a representative sample of the Dutch population [26]. The survey started in 2008 and is conducted on behalf of the Dutch ministry of security and justice, Statistics Netherlands, municipalities and police. The IVM survey uses self-administrating via a pen-and-paper or web-based questionnaire. The IVM 2011 survey was conducted from August 2011 to December 2011. The survey sample of the IVM 2011 consisted of approximately 220,000 people [28]. Since crime victimisation figures vary across geographic regions [29], [30], the IVM 2011 data were matched with the SMI outpatient interviews for geographic regions on the basis of postal code. Dutch postal code areas cover (a part of) an individual neighbourhood and range from 1.1 km2 in large cities to 8.3 km2 in rural areas. A Dutch postal code area houses approximately 4,000 inhabitants [31]. The IVM 2011 sample used as the comparison group consisted of 38,227 people.

### Statistical Analysis

Annual prevalence rates were reported of crime victimisation and of single (1 incident), multiple (2–3 incidents), poly-victimisation (4 or more incidents), and the incident rate of the total sample of SMI outpatients, and stratified by sex. For comparison with the general population, prevalence and incident rates were directly weighted by sex (non-stratified analyses), age, ethnicity and educational level to resemble the distribution of the IVM 2011 sample, as crime victimisation is related to these characteristics in the general population [29], [30]. Since the IVM database did not contain direct information on a person’s source of income (welfare or labour), occupation or salary, it is common practice to use educational level as a proxy for socioeconomic status [32]. Following the definition of the Dutch government [33], ethnicity was classified on the basis of the patient’s country of birth and that (or those) of their parents. Logistic regression analyses were conducted to compare recent crime-victimisation prevalence between the SMI outpatients and the general population, and between male and female SMI outpatients. Bootstrapping was used to obtain the 95% confidence intervals of the prevalence rates. Poisson regression analyses were conducted to compare incident rates for these groups. Sensitivity analyses were performed to assess the reliability of the incident rate, although we excluded four male outpatients who reported extremely high numbers of incidents (i.e. almost daily victimisation). The crime categories reported are identical to those of the IVM reports. We also report crime victimisation categories from which car-related crime (car theft, theft from car and vandalism of car) is excluded, since car ownership is less common among SMI outpatients (27% in our sample versus 89% in the IVM 2011 sample).

## Results

### Sample

The sample consisted of 956 SMI patients: 608 men (64%) and 348 women (36%). Mean age was 44.7 year (SD = 10.4). A majority of respondents (61%) had Dutch ethnicity. Educational level was categorised into no/primary education (23%), basic vocational education (34%), intermediate vocational or preparatory academic education (28%), and high vocational or academic education (15%). While most patients were receiving social welfare (86%), 14% were employed (in regular and/or sheltered employment). Their salaries were mostly low; 68% earned less than the minimum monthly wage of €1,500. The respondents’ demographic characteristics and yearly admittance rates were consistent with the nationwide figures for SMI patients in the Netherlands [24]. Patients’ records showed that 77% of the patients were diagnosed with a chronic psychotic disorder and 23% of the patients with a chronic mood disorder. In the interview 27% of the patients reported alcohol abuse over the past 6 months, i.e. regularly consuming six alcohol drinks or more per day, and 26% of the patients admitted using some kind of drug over the past 12 months. Table 1 presents the baseline sociodemographic characteristics of the sample.

**Table 1:** Respondents’ sociodemographic characteristics.

Characteristic	Subtype	Result (%)
Sex	Male	608 (63.6%)
	Female	348 (36.4%)
Age	m (sd)	44.7 (10.4)
Ethnicity	Dutch	587 (61.4%)
	Surinamese	105 (11.0%)
	Turkish	40 (4.2%)
	Moroccan	43 (4.5%)
	Indonesian	33 (3.5%)
	Antillean	30 (3.1%)
	Other	116 (12.1%)
	Unknown	2 (0.2%)
Marital Status	Single	620 (64.8%)
	Married/cohabiting	170 (17.8%)
	Divorced	157 (16.5%)
	Widow/widower	9 (0.9%)
Living Arrangements	One-person household	489 (51.2%)
	With family	242 (25.3%)
	With friends	8 (0.8%)
	Community or halfway housing	185 (19.4%)
	Unknown	32 (3.3%)
Educational level	No education or primary education	217 (22.7%)
	Basic vocational education	324 (33.9%)
	Intermediate vocational education	268 (28.0%)
	Higher vocational or academic education	147 (15.4%)
Income	Receiving welfare	817 (85.5%)
	In employment	139 (14.5%)
Salary	< €1,500	94 (67.6%)
	€1,500– €2,100	25 (18.0%)
	€2,100– €3,000	10 (7.2%)
	> €3,000	10 (7.2%)
Committed to psychiatric hospital (past year)	Yes	171 (17.9%)
Imprisoned (past year)	Yes	24 (2.5%)

### Annual Prevalence

Tables 2 and 3 report the annual prevalence rates (%) of crime victimisation in the general population, and the unweighted and weighted annual prevalence rates in SMI outpatients.

**Table 2:** SMI Outpatients and the annual weighted and unweighted prevalences of crime victimisation and polyvictimisation (%) relative to the prevalences in the general population.

		SMI Outpatients		General Population	
		Unweighted	Weighted ‡		
		(N = 956)		(N = 38,227)	
Victimisation per type of crime	Abs.	Annual Prevalence	Annual Prevalence	Annual Prevalence	Relative Risk #
		(95% CI)	(95% CI)	(95% CI)	(95% CI)
Property Crime^a^	268	28.0 (25.4–30.6)	23.6 (18.2–26.4)	15.9 (15.4–16.2)	1.48 (1.32–1.67)*
Property Crime, excluding car-related crime^b^	265	27.7 (25.1–30.3)	23.4 (17.9 −25.9)	13.7 (13.3–14.0)	1.70 (1.52–1.92)*
Attempted burglary	37	3.9 (2.7–5.1)	4.2 (2.9–5.5)	2.6 (2.3–2.7)	1.63 (1.19–2.22)*
Burglary	96	10.0 (8.2–12.0)	7.9 (6.3–9.6)	1.5 (1.3–1.7)	5.20 (4.13–6.55)*
Bicycle theft	91	9.5 (7.7–11.4)	8.4 (6.7–10.1)	5.8 (5.4–6.0)	1.45 (1.17–1.80)*
Car theft	1	0.1 (0.0–0.4)	0.1 (0.0–0.3)	0.7 (0.5–0.7)	0.16 (0.02–1.12)
Car owners only †	1	0.4 (0.0–1.2)	0.3 (0.0–1.0)	0.7 (0.5–0.8)	0.43 (0.06–3.08)
Theft from car	2	0.2 (0.0–0.5)	0.3 (0.0–1.8)	2.6 (2.4–2.8)	0.04 (0.06–0.29)*
Car owners only †	2	0.8 (0.0–2.0)	0.3 (0.0–1.0)	2.9 (2.6–3.1)	0.11 (0.02–0.79)*
Vandalism of car	33	3.3 (2.3–4.8)	4.4 (3.2–5.9)	12.0 (11.5–12.3)	0.37 (0.27–0.49)*
Car owners only †	33	12.7 (8.7–17.1)	13.5 (10.0–17.4)	13.4 (12.9–13.7)	1.01 (0.76–1.34)
Pick-pocketing	40	4.2 (2.9–5.5)	3.1 (2.1–4.3)	1.9 (1.6–2.0)	1.68 (1.17–2.41)*
Robbery	11	1.2 (0.5–1.9)	0.5 (0.1–1.0)	0.3 (0.3–0.4)	1.56 (0.64–3.80)
Theft (other)	64	6.7 (5.1–8.3)	5.5 (4.1–7.1)	4.2 (3.8–4.4)	1.32 (1.02–1.73)*
Vandalism (other)	101	10.6 (8.6–12.4)	9.7 (7.8–11.6)	7.7 (7.3–8.0)	1.26 (1.03–1.53)*
Vandalism^c^	132	13.8 (11.6–16.1)	14.1 (12.0–16.6)	17.6 (17.2–18.0)	0.80 (0.68–0.94)*
Personal Crime^d^	183	19.1 (16.8–21.7)	17.1 (14.6–19.6)	6.1 (5.7–6.3)	2.81 (2.43–3.25)*
Sexual harassment or assault	52	5.4 (4.1–6.9)	6.4 (4.8–8.1)	1.6 (1.3–1.7)	3.94 (3.05–5.09)*
Threats of violence	106	11.1 (9.2–13.1)	8.9 (7.2–10.9)	4.2 (3.8–4.4)	2.12 (1.72–2.61)*
Physical assault	61	6.4 (4.9–8.1)	5.6 (4.3–7.1)	1.1 (1.0–1.3)	4.85 (3.69–6.39)*
Crime (other)	45	4.7 (3.3–6.1)	5.0 (3.7–6.5)	1.3 (1.1–1.4)	3.82 (2.86–5.10)*
Total crime^e^	445	46.5 (43.4–49.6)	43.2 (35.0–46.0)	32.0 (31.4–32.4)	1.35 (1.25–1.46)*
Total Crime, excluding car-related crime^f^	423	44.2 (41.0–47.3)	40.5 (32.5–43.3)	23.3 (22.8–23.8)	1.74 (1.60–1.88)*

a Comprises burglary, attempted burglary, bicycle theft, car theft, theft from car, pick-pocketing, robbery, theft (other).

b Comprises burglary, attempted burglary, bicycle theft, pick-pocketing, robbery, theft (other).

c Comprises vandalism of car, vandalism (other).

d Comprises sexual harassment or assault, threats of violence, physical assault.

e Comprises burglary, attempted burglary, bicycle theft, car theft, theft from car, car vandalism, pick-pocketing, robbery, theft (other), vandalism (other), sexual harassment or assault, threats of violence, physical assault, crime (other).

f Comprises burglary, attempted burglary, bicycle theft, pick-pocketing, robbery, theft (other), vandalism (other), sexual harassment or assault, threats of violence, physical assault, crime (other).

*p<.05.

† Car owners in unweighted sample (N = 260); Car owners in weighted sample (N = 310); Car owners in matched IVM 2011 sample (N = 34,161).

# SMI outpatient sample weighted for sex, age, ethnicity, and educational level; IVM 2011 sample matched by region. The general population serves as a reference category.

‡ Weighted for sex, age, ethnicity and educational level.

**Table 3:** SMI Outpatients and the annual weighted and unweighted prevalences of crime polyvictimisation (%) relative to the prevalences in the general population.

		SMI Outpatients			General Population	
		Unweighted		Weighted ‡		
		(N = 956)			(N = 38,227)	
Victimisationper type of crime	Number ofincidents	Abs.	AnnualPrevalence	AnnualPrevalence	AnnualPrevalence	RelativeRisk #
			(95% CI)	(95% CI)	(95% CI)	(95% CI)
Total Crime, excludingcar-related crime^a^	1 incident	182	19.1 (16.8–21.7)	17.4 (15.0–19.9)	13.4 (12.9 −13.7)	1.30 (1.13–1.50)*
	2–3 incidents	138	14.5 (12.3–16.7)	13.0 (10.9–15.2)	7.4 (6.9–7.6)	1.77 (1.49–2.09)*
	4 or more incidents	102	10.7 (8.9–12.9)	9.9 (8.1–11.8)	2.2 (1.9–2.4)	4.47 (3.66–5.48)*
Property Crime, excludingcar-related crime^b^	1 incidents	166	17.4 (15.1–19.8)	14.6 (12.5–17.0)	9.7 (9.3–10.0)	1.51 (1.29–1.76)*
	2–3 incidents	64	6.7 (5.1–8.4)	5.9 (4.4–7.4)	3.2 (2.9–3.4)	1.82 (1.41–2.37)*
	4 or more incidents	34	3.6 (2.4–4.8)	2.4 (1.5–3.5)	0.5 (0.4–0.5)	4.89 (3.12–7.51)*
Personal Crime^c^	1 incident	103	10.8 (8.9–12.7)	9.2 (7.3–11.2)	3.8 (3.4–3.9)	2.45 (1.99–3.01)*
	2–3 incidents	40	4.2 (2.9–5.6)	4.0 (2.8–5.2)	1.8 (1.5–2.0)	2.17 (1.58–2.99)*
	4 or more incidents	41	4.3 (3.0–5.6)	4.0 (2.8–5.2)	0.4 (0.3–0.4)	10.85 (7.63–15.44)*

a Comprises burglary, attempted burglary, bicycle theft, pick-pocketing, robbery, theft (other), vandalism (other), sexual harassment or assault, threats of violence, physical assault, crime (other).

b Comprises burglary, attempted burglary, bicycle theft, pick-pocketing, robbery, theft (other).

c Comprises sexual harassment or assault, threats of violence, physical assault.

*p<.05.

# SMI outpatient sample weighted for sex, age, ethnicity, and educational level; IVM 2011 sample matched by region. The general population serves as a reference category.

‡ Weighted for sex, age, ethnicity and educational level.

The annual prevalence rate of victimisation in the SMI outpatients in our sample was 46.5%, against 32% in the general population. SMI outpatients were most commonly the victims of threats of violence, vandalism (other than car related), and burglary. Relative to the general population, an SMI outpatient had a 1.35 relative risk of being the victim of a crime over a 12-month period. The highest prevalence rates in the general population were observed for vandalism of a car, of other forms of vandalism, and of being threatened with violence.

Relative to the general population, SMI outpatients suffered high prevalence rates for compounded property crime and personal crime categories. Regarding property crimes, higher crime prevalence rates were found for burglary, attempted burglary, pick-pocketing, theft (other), and vandalism (other). There were no significant differences with regard to car theft and robbery. SMI outpatients reported less car theft or car vandalism. Regarding personal crime, prevalence rates were significantly higher for SMI outpatients than for the general population for all three subcategories: i.e. sexual harassment or assault, threatened with violence and physical assault. The prevalence rate for personal crime in SMI outpatients was 2.8 times higher than in the general population.

Over the previous 12 months, over 10% of the SMI outpatients had been victims of more than 4 crime incidents. Poly-victimisation prevalence rates for property crime were 3.6%; for personal crime they were 4.3%. Relative risk of poly-victimisation of SMI outpatients compared to the general population was 4.89 for property crime and 10.85 for personal crime.

### Annual Prevalence by Sex

Tables 4 and 5 show the annual victimisation prevalence rates for male and female SMI outpatients, and compares them with those for men and women in the general population. The annual prevalence rates for male and female SMI outpatients differed little. The relative risk showed that SMI women were three times more likely than SMI men to fall victim to sexual harassment or assault. Male and female SMI outpatients reported a higher overall prevalence rate than men and women in the general population. The relative rates for total victimisation were 1.27 for men and 1.43 for women.

**Table 4:** Male and female SMI outpatients and the prevalences of annual crime victimisation (%) relative to the prevalences in the general population.

	Men		Women		
	Annual Prevalence ¥	RR relative to men inthe general population #	AnnualPrevalence ¥	RR relative towomen in thegeneral population #	RR male relative to femaleSMI outpatients ¥
	(95% CI)	(95% CI)	(95% CI)	(95% CI)	(95% CI)
Victimisationper type of crime	Men (N = 608)	Men (N = 17,494)	Women(N = 348)	Women(N = 20,733)	
Property Crime^a^	28.8 (25.2–32.4)	1.49 (1.25–1.76)*	26.7 (22.4–31.6)	1.48 (1.26–1.73)*	1.08 (.87–1.33)
Property Crime, excludingcar-related crime^b^	28.3 (24.8–31.7)	1.74 (1.47–2.08)*	26.7 (22.4–31.6)	1.67 (1.42–1.95)*	1.06 (.85–1.31)
Attempted burglary	3.5 (2.1 −5.1)	0.9 (0.53 −1.71)	4.6 (2.6–6.9)	2.23 (1.55–3.21)*	0.75 (0.40–1.42)
Burglary	11.2 (8.9–13.8)	5.76 (4.16–7.99)*	8.0 (5.2–10.9)	4.74 (3.42–6.55)*	1.39 (0.91–2.12)
Bicycle theft	9.7 (7.6–12.2)	1.38 (1.00–1.90)*	9.2 (6.3 −12.1)	1.51 (1.14–2.01)*	1.06 (0.70–1.59)
Car theft §	0.2 (0.0–0.5)	0.27 (0.04–1.90)	–	–	–
Car owners only †	0.7 (0.0–2.4)	0.91 (0.13–6.43)	–	–	–
Theft from car§	0.3 (0.0–0.8)	0.07 (0.01–0.51)*	–	–	–
Car owners only†	1.4 (0.0–3.7)	0.25 (0.04–1.73)	–	–	–
Vandalism of car	3.0 (1.6–4.3)	0.22 (0.13–0.38)*	4.3 (2.3–6.6)	0.51 (0.36–0.72)*	0.69 (0.35–1.35)
Car owners only†	12.5 (6.9–18.7)	0.74 (0.43–1.27)	12.9 (6.9–19.4)	1.20 (0.86–1.67)	0.97 (0.51–1.83)
Pick-pocketing	3.6 (2.1–5.3)	1.93 (1.09–3.42)*	5.2 (2.9–7.5)	1.46 (0.91–2.35)	0.70 (0.38–1.29)
Robbery	1.3 (0.5–2.3)	1.16 (0.29–4.72)	0.9 (0.0–2.0)	1.35 (0.33–5.52)	1.53 (0.41–5.72)
Theft (other)	7.4 (5.3–9.4)	1.76 (1.24–2.51)*	5.5 (3.2–8.0)	1.00 (0.67–1.50)	1.36 (0.81–2.28)
Vandalism (other)	10.9 (8.4–13.3)	1.34 (1.02–1.76)*	10.1 (6.9–13.5)	1.19 (0.90–1.57)	1.08 (0.73–1.59)
Vandalism^c^	13.5 (10.7–16.3)	0.74 (0.58–0.94)*	14.4 (10.6–18.1)	0.86 (0.69–1.06)	0.94 (0.68–1.30)
Personal Crime^d^	18.3 (15.1–21.4)	2.51 (2.02–3.13)*	20.7 (16.4–25.3)	3.09 (2.54–3.74)*	0.88 (0.68–1.15)
Sexual harassmentor assault	3.0 (1.8–4.4)	5.92 (3.59–9.76)*	9.8 (6.6–12.9)	3.56 (2.66–4.77)*	0.30 (0.17–0.53)*
Threats of violence	12.3 (9.7–15.0)	1.97 (1.49–2.61)*	8.9 (5.7–12.1)	2.33 (1.71–3.18)*	1.39 (0.93–2.06)
Physical assault	6.4 (4.6–8.4)	4.05 (2.72–6.05)*	6.3 (4.0–9.2)	5.84 (3.99–8.54)*	1.02 (0.61–1.68)
Crime (other)	3.9 (2.5–5.8)	2.09 (1.23–3.55)*	6.0 (3.7–8.3)	5.79 (4.09–8.21)*	0.65 (0.37–1.16)
Total crime^e^	45.7 (41.9–49.7)	1.27 (1.13–1.42)*	48.0 (42.8–53.2)	1.43 (1.29–1.57)*	0.95 (0.83–1.10)
Total Crime, excludingcar-related crime^f^	43.4 (39.6–47.4)	1.65 (1.46–1.86)*	45.7 (40.5–50.9)	1.81 (1.63–2.00)*	0.95 (0.82–1.10)

a Comprises burglary, attempted burglary, bicycle theft, car theft, theft from car, pick-pocketing, robbery, theft (other).

b Comprises burglary, attempted burglary, bicycle theft, pick-pocketing, robbery, theft (other).

c Comprises vandalism of car, vandalism (other).

d Comprises sexual harassment or assault, threats of violence, physical assault.

e Comprises burglary, attempted burglary, bicycle theft, car theft, theft from car, car vandalism, pick-pocketing, robbery, theft (other), vandalism (other), sexual harassment or assault, threats of violence, physical assault, crime (other).

f Comprises burglary, attempted burglary, bicycle theft, pick-pocketing, robbery, theft (other), vandalism (other), sexual harassment or assault, threats of violence, physical assault, crime (other).

*p<.05.

† Male car owners in unweighted sample (N = 144); Female car owners in unweighted sample (N = 116); Male car owners in matched IVM 2011 sample (N = 15,786); Female car owners in matched IVM 2011 sample (N = 18,375).

# SMI outpatient sample weighted for age, educational level and ethnicity; IVM 2011 sample matched by region.

§ As female SMI outpatients reported no incidents of car theft or theft from car, prevalence rates and relative rate ratios could not be calculated.

¥ Unweighted data.

**Table 5:** Male and female SMI outpatients and the prevalences of annual crime polyvictimisation (%) relative to the prevalences in the general population.

		Men		Women		
		Annual Prevalence ¥	RR relative to menin the generalpopulation #	Annual Prevalence ¥	RR relative to womenin thegeneral population #	RR male relative tofemaleSMI outpatients ¥
		(95% CI)	(95% CI)	(95% CI)	(95% CI)	(95% CI)
Type ofcrime	Number ofincidents	Men(N = 608)	Men(N = 17,494)	Women(N = 348)	Women(N = 20,733)	
Total Crime,excludingcar-relatedcrime^a^	1 incident	18.8 (15.8–22.0)	1.22 (0.98–1.51)	19.5 (15.5–23.9)	1.35 (1.12–1.63)*	.96 (.73–1.26)
	2–3 incidents	15.3 (12.5–18.3)	1.85 (1.46–2.36)*	12.9 (9.4–16.7)	1.66 (1.31–2.10)*	1.18 (.85–1.65)
	4 or moreincidents	9.4 (6.9–11.7)	3.82 (2.78–5.26)*	12.9 (9.5–16.7)	5.04 (3.88–6.55)*	.73 (.50–1.05)
Property Crime,excludingcar-relatedcrime^b^	1 incident	18.1 (15.1–21.2)	1.58 (1.25–1.98)*	16.1 (12.4–19.8)	1.45 (1.17–1.79)*	1.12 (.84–1.51)
	2–3 incidents	6.7 (4.8–8.9)	1.93 (1.32–2.82)*	6.6 (4.0–9.5)	1.74 (1.22–2.48)*	1.02 (.62–1.67)
	4 or moreincidents	3.5 (2.0–5.1)	4.60 (2.41–8.77)*	3.7 (2.0–5.8)	4.74 (2.62–8.56)*	.93 (.47–1.82)
Personal Crime^c^	1 incident	10.5 (8.4–13.0)	2.22 (1.63–3.00)*	11.2 (8.1–14.7)	2.68 (2.03–3.54)*	.94 (.65–1.37)
	2–3 incidents	4.0 (2.5–5.6)	1.81 (1.09–3.01)*	4.6 (2.6–6.9)	2.49 (1.65–3.76)*	.86 (.46–1.59)
	4 or moreincidents	4.0 (2.5–5.6)	10.80 (6.38–18.30)*	4.9 (2.9–7.2)	10.87 (6.77–17.48)*	.81 (.44–1.48)

a Comprises burglary, attempted burglary, bicycle theft, pick-pocketing, robbery, theft (other), vandalism (other), sexual harassment or assault, threats of violence, physical assault, crime (other).

b Comprises burglary, attempted burglary, bicycle theft, pick-pocketing, robbery, theft (other).

c Comprises sexual harassment or assault, threats of violence, physical assault.

*p<.05.

# SMI outpatient sample weighted for age, educational level and ethnicity; IVM 2011 sample matched by region.

¥ Unweighted data.

### Annual Incident Rate

Table 6 shows the annual incident rates of crime victimisation in SMI outpatients, and compares with those in the general population. SMI outpatients reported 2,687 crimes per 1,000 patients per year. The highest ranking crimes among SMI outpatients were 1.) sexual harassment or sexual assault; and 2.) threats of violence, in both of which there were over 800 annual incidents per 1,000 persons. The highest ranking incidents in the general population are car-related vandalism (168 incidents per 1,000 persons).

**Table 6:** SMI Outpatients and the weighted and unweighted annual incident rates of crime victimisation relative to the rates in the general population.

	SMI Outpatients		General Population	
	Unweighted	Weighted ‡		
	(N = 956)		(N = 38,227)	
	3,415 Incidents	2,536 Incidents	24,266 Incidents	
Victimisation per type of crime	Incident rate per 1,000 SMI outpatients	Incident rate per 1,000 SMI outpatients	Incident rate per 1,000 persons	IRR #
	(95% CI)	(95% CI)	(95% CI)	(95% CI)
Property Crime^a^	976 (953–999)	703 (688–721)	234 (229–239)	3.01 (2.78–3.26)*
Property Crime, excluding car-related crime^b^	971 (945–997)	701 (684–721)	194 (190–199)	3.62 (3.34–3.92)*
Attempted burglary	132 (125–139)	123 (117–131)	32 (30–34)	3.88 (3.22–4.69)*
Burglary	179 (166–193)	150 (139–162)	18 (17–19)	8.33 (6.95–9.97)*
Bicycle theft	129 (124–134)	109 (105–113)	71 (68–73)	1.54 (1.27–1.88)*
Car theft	1 (–)	1 (–)	8 (7–9)	0.13 (0.02–0.90)*
Car owners only †	4 (3–4)	3 (3–4)	9 (8–10)	0.35 (0.05–2.48)
Theft from car	4 (–)	2 (–)	32 (30–33)	0.07 (0.02–0.27)*
Car owners only †	15 (14–15)	6 (6–7)	35 (33–37)	0.18 (0.05–0.73)*
Vandalism of car	58 (56–59)	64 (62–66)	168 (164–172)	0.38 (0.30–0.49)*
Car owners only †	212 (206–217)	197 (192–202)	188 (184–193)	1.04 (0.81–1.34)
Pick-pocketing	68 (63–73)	66 (61–71)	18 (17–20)	3.61 (2.79–4.67)*
Robbery	24 (20–28)	13 (11–15)	4 (3–5)	3.25 (1.81–5.86)*
Theft (other)	441 (421–463)	248 (237–260)	51 (49–54)	4.84 (4.23–5.54)*
Vandalism (other)	205 (198–212)	188 (182–195)	115 (112–118)	1.64 (1.41–2.55)*
Vandalism^c^	263 (257–268)	252 (248–258)	283 (278–289)	0.89 (0.78–1.01)
Personal Crime^d^	1822 (1766–1881)	1379 (1339–1426)	101 (98–104)	13.68 (12.85–14.56)*
Sexual harassment or assault	889 (830–942)	673 (633–718)	25 (23–27)	26.96 (24.39–29.79)*
Threats of violence	827 (794–862)	613 (590–640)	61 (58–63)	10.13 (9.25–11.09)*
Physical assault	111 (102–120)	93 (86–101)	15 (14–17)	6.07 (4.86–7.59)*
Crime (other)	512 (473–553)	354 (328–383)	16 (15–18)	21.56 (18.89–24.61)*
Total crime^e^	3572 (3521–3625)	2687 (2654–2732)	635 (627–643)	4.24 (4.07–4.42)*
Total Crime, excludingcar-related crime^f^	3509 (3442–3578)	2621 (2576–2678)	427 (420–433)	6.16 (5.90–6.42)*

a Comprises burglary, attempted burglary, bicycle theft, car theft, theft from car, pick-pocketing, robbery, theft (other).

b Comprises burglary, attempted burglary, bicycle theft, pick-pocketing, robbery, theft (other).

c Comprises vandalism of car, vandalism (other).

d Comprises sexual harassment or assault, threats of violence, physical assault.

e Comprises burglary, attempted burglary, bicycle theft, car theft, theft from car, car vandalism, pick-pocketing, robbery, theft (other), vandalism (other), sexual harassment or assault, threats of violence, physical assault, crime (other).

f Comprises burglary, attempted burglary, bicycle theft, pick-pocketing, robbery, theft (other), vandalism (other), sexual harassment or assault, threats of violence, physical assault, crime (other).

*p<.05.

† Car owners in unweighted sample (N = 260); Car owners in weighted sample(N = 310); Car owners in matched IVM 2011 sample (N = 34,161).

# SMI outpatient sample weighted for sex, age, ethnicity, and educational level; IVM 2011 sample matched by region. The general population serves as a reference category.

‡ Weighted for sex, age, ethnicity and educational level.

The overall crime incident rates were higher for SMI outpatients than for the general population, with significantly higher incident-rate ratios in almost all unique crime categories. One exception involved car-related crime incidents, but this can be explained by the limited number of car owners among SMI outpatients. IRR for property crimes was over three times higher for SMI outpatients than for the general population. Relative to the incident rate in the general population, the incident rate of burglary in SMI outpatients was over eight times higher. Over 13 times more personal crime incidents were reported by SMI outpatients than by the general population. Sexual harassment and assault incidents were reported 27 times more by SMI outpatients than by people in the general population.

The incident rates of SMI outpatients were lower after the removal from the analyses of the outpatients who had reported daily victimisation; this reduced the overall crime-incident rate by 36%. The greatest reduction was for sexual harassment or assaults (66%). IRRs remained significantly higher in all unique crime categories, including sexual harassment or assaults (alternative IRR = 9.25; 95% CI = 7.99–10.71).

### Annual Incident Rate by Sex

Table 7 shows the annual incident rates for male and female SMI outpatients, and compares them with those for men and women in the IVM sample. Male SMI outpatients reported 4378 crimes per 1000 patients per year, and female outpatients reported 2163. Male and female SMI outpatients reported more incidents in all unique crime categories than men and women in the general population. Sexual harassment or assaults were reported more than 100 times more often by male SMI outpatients than by men in the general population. After removal from the analyses of the four male outpatients who reported being victimised daily, the incident rate of male SMI outpatients fell by 83% for sexual harassment or assaults (alternative IRR = 17.7; 95% CI = 13.7–23.0); and by 58% for total crime (alternative IRR = 2.4; 95% CI = 2.2–2.6).

**Table 7:** Male and female SMI outpatients and the annual incident rate of crime victimisation relative to that in the general population.

	Men (N = 608)		Women (N = 348)		
	2,662 Incidents		753 Incidents		
Victimisation pertype of crime	Incident rate per 1,000SMI outpatients ¥	IRR relative togeneralpopulation #	Incident rateper 1,000 SMIoutpatients ¥	IRR relative togeneral population #	IRR male relative tofemale SMI outpatients ¥
	(95% CI)	(95% CI)	(95% CI)	(95% CI)	(95% CI)
Property Crime^a^	1092 (969–1231)	3.28 (2.93–3.66)*	773 (686–871)	2.76 (2.47–3.08)*	1.41 (1.23–1.63)*
Property Crime,excludingcar-relatedcrime^b^	1084 (962–1221)	4.11 (3.67–4.60)*	773 (686–871)	3.20 (2.86–3.58)*	1.40 (1.22–1.62)*
Attempted burglary	97 (76–123)	1.78 (1.20–2.64)*	193 (152–245)	5.85 (4.70–7.27)*	0.50 (0.36–0.72)*
Burglary	173 (136–220)	7.16 (5.39–9.52)*	190 (149–241)	9.23 (7.31–11.65)*	0.91(0.67–1.24)
Bicycle theft	127 (95–169)	1.42 (1.05–1.92)*	132 (99–176)	1.64 (1.27–2.12)*	0.96 (0.66–1.38)
Car theft§	2 (0–12)	0.21 (0.03–1.52)	–	–	–
Car owners only†	7 (1–49)	0.72 (0.10–5.17)	–	–	–
Theft from car §	7 (2–18)	0.12 (0.03–0.48)*	–	–	–
Car owners only†	28 (10–74)	0.40 (0.10–1.62)	–	–	–
Vandalism of car	38 (49–99)	0.18 (0.11–0.30)*	92 (65–130)	0.56 (0.42–0.75)*	0.76 (0.59–0.97)*
Car owners only	160 (113–226)	0.61 (0.36–1.03)	276 (195–390)	1.34 (1.01–1.79)*	0.58 (0.34–0.99)*
Pick-pocketing	43 (31–59)	3.03 (1.88–4.89)*	112 (82–153)	3.88 (2.85–5.26)*	0.38 (0.23–0.63)*
Robbery	18 (10–32)	1.54 (0.49–4.88)	34 (20–61)	5.13 (2.56–10.26)*	0.52 (0.23–1.19)
Theft (other)	627 (461–851)	9.14 (7.81–10.69)*	118 (87–160)	1.63 (1.21–2.19)*	5.32 (3.85–7.34)*
Vandalism (other)	197 (158–247)	1.71 (1.38–2.12)*	218 (174–273)	1.57 (1.27–1.93)*	0.90 (0.68–1.20)
Vandalism^c^	235 (195–284)	0.79 (0.65–0.96)*	310 (257–348)	0.98 (0.82–1.16)	0.76 (0.24–0.70)*
Personal Crime^d^	2375 (2120–2661)	21.22 (19.64–22.93)*	856 (764–959)	6.72 (5.99–7.54)*	2.77 (2.45–3.14)*
Sexual harassmentor assault	1164 (985–1377)	107.18 (90.68–126.67)*	394 (333–465)	7.11 (5.93–8.53)*	2.96 (2.46–3.55)*
Threats of violence	1118 (929–1347)	13.14 (11.80–14.64)*	319 (265–384)	5.98 (5.03–7.11)*	3.51 (2.87–4.29)*
Physical assault	92 (70–122)	4.13 (2.88–5.91)*	144 (109–190)	8.33 (6.25–11.11)*	0.64 (0.44–0.94)*
Crime (other)	676 (541–844)	24.23 (20.37–28.83)*	224 (180–280)	18.30 (14.91–22.45)*	3.02 (2.37–3.84)*
Total crime^e^	4378 (4076–4702)	5.64 (5.35–5.94)*	2164 (2015–2324)	2.95 (2.77–3.16)*	2.02 (1.87–2.19)*
Total Crime,excluding car-related crime^f^	4332 (4027–4660)	8.52 (8.07–8.99)*	2072 (1926–2229)	4.09 (3.82–4.38)*	2.09 (1.93–2.27)*

a Comprises burglary, attempted burglary, bicycle theft, car theft, theft from car, pick-pocketing, robbery, theft (other).

b Comprises burglary, attempted burglary, bicycle theft, pick-pocketing, robbery, theft (other).

c Comprises vandalism of car, vandalism (other).

d Comprises sexual harassment or assault, threats of violence, physical assault.

e Comprises burglary, attempted burglary, bicycle theft, car theft, theft from car, car vandalism, pick-pocketing, robbery, theft (other), vandalism (other), sexual harassment or assault, threats of violence, physical assault, crime (other).

f Comprises burglary, attempted burglary, bicycle theft, pick-pocketing, robbery, theft (other), vandalism (other), sexual harassment or assault, threats of violence, physical assault, crime (other).

*p<.05.

† Male car owners in unweighted sample (N = 144); Female car owners in unweighted sample (N = 116); Male car owners in matched IVM 2011 sample (N = 15,786); Female car owners in matched IVM 2011 sample (N = 18,375).

# SMI outpatient sample weighted for age, educational level and ethnicity; IVM 2011 sample matched by region.

§ As female SMI outpatients reported no incidents of car theft or theft from car, no incidence rates and incidence rate ratios could be calculated.

¥ Unweighted data.

## Discussion

In this multisite epidemiological survey on crime victimisation among 956 psychiatric patients with SMI who were being treated in outpatient MHC, we report 12-month prevalence and incident rates, and compare these rates with those for crime victimisation in the general population.

### High Prevalence and Incident Rates for SMI Patients

There were high prevalence and incident rates for victimisation in psychiatric patients. Individual SMI outpatients had a 1.35 times greater risk of being a crime victim than individuals in the general population, and were subject to 4.24 times more incidents.

This higher risk in SMI outpatients applied in all crime categories. SMI outpatients were the most vulnerable to being victims of personal crimes, i.e. sexual harassments and assaults, threats of violence, and physical assaults. Relative to the general population, the SMI outpatients were 2.81 more likely to become victims of a personal crime for all categories; the particular risk of sexual harassment was 3.94 higher. As they had experienced 4 incidents or more over the past year, approximately 10% of SMI outpatients can be categorised as poly-victims.

Gender differences in victimisation patterns among SMI outpatients showed that, relative to their male counterparts, female SMI outpatients had a three times greater annual risk of becoming victims of sexual harassment or sexual assault. Otherwise, except for sexual crimes, the prevalence rates between male and female outpatients did not differ significantly. However, male SMI outpatients reported twice as many incidents as females – particularly higher incident rates of sexual crimes, threats of violence, and theft. Sensitivity analyses showed that these high incident rates can be explained by the existence of a subpopulation of male outpatients with high to extremely high incident rates of crime victimisation.

In the general population, men have higher prevalence and incident rates for personal victimisation than women. For sexual crimes, however, the prevalence and incident rates in the general population were lower for men than for women [29], [30], [34]. In our sample, we found no effects of gender on victimisation. Instead, female and male SMI outpatients reported similar rates of personal victimisation, and male SMI outpatients were vulnerable for sexual crimes to the same extent as female SMI outpatients. The incident rates even suggested that, in certain subpopulations of male SMI outpatients, the extent of sexual victimisation can be more severe than in their female counterparts.

While some previous research has supported these findings [35], other studies found that females were more vulnerable to sexual victimisation, and that males were more at risk of physical victimisation [8], [36], [37]. We speculate that mechanisms which prevent women falling victim to personal victimisation and men to sexual victimisation – mechanisms such as such as lifestyle characteristics, social control or cultural norms – [38] do not apply to SMI patients. A similar change in gender-related victimisation patterns has been observed in prison populations [39] and in sexual minorities [40].

### Results are in Accordance with Previous Studies

Our overall finding that SMI patients are at greater risk of victimisation than people in the general population is consistent with previous studies [1], [9]. However, due to differences in methodology (e.g. recency, study designs, research populations and the operationalisation of victimisation incidents), specific crime-victimisation figures cannot easily be compared across studies. The design of this study resembles that of research by Teplin and colleagues among SMI patients in Chicago [8], in which the prevalence rates of personal crime among SMI outpatients were similar to those in our study: personal crime prevalence is 19% in Dutch patients against 25% in US patients, and property-crime prevalence 28% in both Dutch and US patients. But as the prevalence rate in the US comparison population was much lower than in the Dutch comparison population, the relative rates for SMI patients found by Teplin and colleagues [8] were higher than in our sample: the relative rate in US patients was 11.8 for personal crime and 4.2 for property crime; in Dutch patients, the rates are 2.8 for personal crime and 1.5 for property crime. However, after control for demographic differences, the incident rates in the US study were not as high as those in our study: while Teplin and colleagues [8] reported that the incident rate for personal crime victimisation was 4 times higher in SMI patients than the general population, the incident rate of personal crime in the Dutch sample was 13 times higher than in the general population. They concluded that the fact that the incident rate ratios in their sample were lower than the prevalence rate ratios indicated that the high prevalence of victimisation in SMI patients could not simply be accounted for by a small group of individuals reporting a very high number of events. As we discussed above, among the Dutch SMI patients, a subgroup of patients did report a very high number of incidents. But even when we excluded these patients from our analyses, incident rate ratios in the Dutch sample remained higher than prevalence rate ratios. It is unclear whether this reflects a genuine difference in incident rates between US and Dutch SMI patients – or whether, for example, different social and cultural circumstances meant that Dutch patients had a lower threshold for reporting incidents – or whether it is the result of differences in research methodology.

### Strengths and Limitations

The strengths of our study include the random selection of participating SMI outpatients, the large sample size, and the opportunity to compare SMI outpatient victimisation rates with those in the general Dutch population. Weighting of the sample minimised the effect of sociodemographic and socioeconomic differences.

Our study also has several limitations. First, we have used MHC services as an entry to contact SMI patients. Although mandatory health insurance in the Netherlands means that all Dutch SMI persons can receive MHC, we will have missed those who refused treatment, were homeless or undocumented, none of whom could be reached. For all we know, this group of SMI persons may be particularly vulnerable to victimisation [41], and the figures we provide may therefore underestimate the actual victimisation rate among persons with SMI in the Netherlands.

The same effect may also have resulted from our exclusion of patients who were too aggressive or were suffering from overtly psychotic symptoms, two documented risk factors for victimisation [35], [36], [42]. The second limitation is that, for inclusion, we used information from the EPF, which contains information on the patients’ addresses and on the diagnosis provided by psychiatrists and clinicians. This creates two dilemmas. On the one hand, because we did not conduct a structured clinical interview to obtain diagnostic information, we cannot provide detailed information on the relationship between victimisation risk and diagnosis. On the other hand, not all EPFs were filled out correctly. We tried to prevent the false inclusion or exclusion of patients by having the clinicians check the extracted information from the EPF. Although our non-response analyses did not indicate a systematic bias, we cannot rule out the possibility that we missed certain subpopulations of difficult SMI patients, and thereby underestimated the victimisation figures.

The final limitation is related to the questionnaire and the mode of administration. The IVM survey is a self-report measurement. Therefor we cannot completely rule out under- or over reporting of victimisation, especially among the current study population. Some of our respondents showed overt signs of psychosis, including paranoia (‘being followed by the secret police’). To detect false reports of crime victimisation, interviewers were trained to apply the IVM crime-victimisation scale and to branch to a series of questions about specific details of the crime. Thorough inspection of the crime victimisation data enabled us to remove unreliable interviews from the sample (N = 44). Although it is possible that we did not detect false crime incidents that were reported in a logical and consistent manner, we think it more likely that crime victimisation incidents were underreported. Psychiatric patients have been reported to accept norm-deviating behaviour more easily, and not to recognise certain types of theft or intimidating interactions for what they are [43]. Our comparison group was administered using a pen-and-paper or web-based questionnaire, while the SMI outpatients were interviewed face-to-face. The mode of administration can influence the data quality. Self-administration may increase the respondents’ willingness to disclose sensitive information compared to face-to-face interviews, but the possibility to give a respondent feedback during the interview might result in more detailed answers and higher item-response rates [44]. However, since we did not re-interview or allocate a subset of respondents to a different administration mode, the exact extent of under-reporting or over-reporting and the impact on the comparison with the general population remains unclear.

### Implications and Recommendations

Our results suggest that crime victimisation is a serious problem among Dutch SMI outpatients. Crime victimisation has been linked to psychopathology, including post-traumatic stress disorders, substance abuse, anxiety, and mood disorders [45]–[47]. In psychiatric patients, victimisation is associated with an exacerbation of existing psychopathology, higher service use, and suboptimal treatment results [46], [48], [49]. It is also likely that it increases the risk of re-victimisation and violent perpetration [17], [45], [50], thereby amplifying its negative consequences and creating a cycle of violence in which perpetration and victimisation are both prevalent [21], [41], [46].

The prevalence of poly-victimisation in our sample suggests that this cycle of violence is a reality for at least 10% of SMI patients. To break it, MHC institutions and clinicians should develop and implement treatment strategies that prevent first-time victimisation and re-victimisation. Skills-based programmes can help patients to become aware of victimisation risks, and can provide tools for averting unsafe situations and for knowing how to respond to them [1], [8], [51], [52]. Another strategy is to help the victim cope with the psychological consequences of the crime incident, for instance through treatment of the subsequent PTSD symptoms [53]–[55].

Staff on psychiatric wards or at institutions such as halfway houses often have a falsely optimistic perception of their patients’ safety [43]. This suggests that clinical staff should also become more perceptive of the problem and more alert to it. We strongly recommend that all patients are systematically screened for victimisation risk, and that MHC providers monitor those with a high risk profile (such as patients with co-morbid substance disorders, and those with a history of criminal perpetration and/or (poly)victimisation) [1], [8], [56]). In collaboration with each patient, the MHC providers may then develop a personalised ‘victimisation prevention plan’ that addresses specific risk factors for victimisation.

Future research should address victimisation rates not only among outpatients and inpatients with SMI, but also among psychiatric patients with milder psychopathology, since both treatment setting and psychopathology have been related to victimisation risk [1], [9], [11]. Resilience programmes are important and it is necessary to gain insight into determinants of victimisation. Large-scale longitudinal studies should therefore be conducted to identify predictors of victimisation among SMI patients and to study the consequences of victimisation, including re-victimisation rates and their effects on the course of psychopathological disorders. This information will underlie the development not only of interventions to reduce victimisation, but also of victimisation risk-assessment tools that can be used to screen and monitor SMI patients in the community.

In conclusion, the prevalence and incident rate of crime victimisation is higher among SMI patients than in the general population, and may have serious social and mental-health consequences. MHC institutions and clinicians should be aware of their responsibility for tackling this problem and for providing measures to prevent victimisation.
